# Post-transjugular Intrahepatic Portosystemic Shunt Hepatic Encephalopathy: Sarcopenia Adds Insult to Injury

**DOI:** 10.5152/tjg.2023.21964

**Published:** 2023-04-01

**Authors:** Puja Bhatia Kapoor, Jaya Benjamin, Harshita Tripathi, Yashwant Patidar, Rakhi Maiwall, Guresh Kumar, Yogendra Kumar Joshi, Shiv Kumar Sarin

**Affiliations:** 1 Department of Clinical Nutrition, Institute of Liver and Biliary Sciences, New Delhi, India; 2 Department of Interventional Radiology, Institute of Liver and Biliary Sciences, New Delhi, India; 3 Department of Hepatology, Institute of Liver and Biliary Sciences, New Delhi, India; 4 Department of Clinical Research, Institute of Liver and Biliary Sciences, New Delhi, India

**Keywords:** Ammonia, ascites, nutritional assessment, skeletal muscle density, skeletal muscle mass, variceal bleed

## Abstract

**Background:**

Hepatic encephalopathy, which is a serious complication, and sarcopenia are undesirable consequences in cirrhosis. Transjugular intrahepatic portosystemic shunt increases the risk of hepatic encephalopathy. We investigated the effect of sarcopenia on the incidence of post-transjugular intrahepatic portosystemic shunt hepatic encephalopathy.

**Methods:**

Clinical data of patients who underwent transjugular intrahepatic portosystemic shunt were extracted retrospectively. Computed tomography images at L3 level of scans performed prior to transjugular intrahepatic portosystemic shunt were analyzed to assess skeletal muscle index—expressed as skeletal muscle area (cm^2^)/ height (m^2^).

**Results:**

Of 210 patients who underwent transjugular intrahepatic portosystemic shunt, complete information was available in 79 [male: 68 (86%); age: 50.5 ± 11.2 years; Child–Turcotte–Pugh score: 8.81 ± 1.23; etiology—alcohol: 44 (56%), non-alcoholic steatohepatitis: 16 (20%), others: 19 (24%); transjugular intrahepatic portosystemic shunt indication—ascites: 56 (71%); bleed: 23 (29%); sarcopenics: 42 (53%)]. Post-transjugular intrahepatic portosystemic shunt hepatic encephalopathy developed in 29 (37%) patients. In patients who developed hepatic encephalopathy, both serum ammonia [177.6 ± 82.5 vs. 115.5 ± 40.5 µg/dL, *P*  = .008] and prevalence of sarcopenia [69% vs. 44%; *P*  = .02; odds ratio (95% CI): 2.8 (1.08-7.4), *P*  = .02] were higher, with sarcopenics having 3 times higher risk of hepatic encephalopathy and 8 times higher risk of multiple episode of hepatic encephalopathy [31% vs. 5.4%; odds ratio (95% CI): 8.2 (1.68-40.5), *P*  = .009]. In multivariate analysis, age [odds ratio (95% CI): 1.05 (1.001-1.11), *P*  = .047], serum albumin [odds ratio (95% CI): 0.162 (0.05-0.56), *P*  = .004], and skeletal muscle index [odds ratio (95% CI): 0.925 (0.89-0.99), *P*  = .017] were independently associated with post-transjugular intrahepatic portosystemic shunt hepatic encephalopathy.

**Conclusions:**

Sarcopenia is present in nearly half of the cirrhotic patients undergoing transjugular intrahepatic portosystemic shunt, which increases the risk of a single episode of hepatic encephalopathy by 3-fold and that of multiple episodes of hepatic encephalopathy by 8-fold after transjugular intrahepatic portosystemic shunt procedure. Increased skeletal muscle index is associated with decreased risk of hepatic encephalopathy.

**Main Points**

- This retrospective study highlights a high prevalence (50%) of sarcopenia in patients with cirrhosis at the time of undergoing the transjugular intrahepatic portosystemic shunt (TIPS) procedure.
- This pre-TIPS sarcopenia significantly increases the risk of developing both single and multiple episodes of post-TIPS hepatic encephalopathy (HE).
- Low muscle mass does predict post-TIPS HE, thus highlighting a careful screening of patients from a nutritional angle before performing the TIPS procedure.

## Introduction

Patients with cirrhosis having the complications of recurrent variceal hemorrhage and ascites that are refractory to standard treatment often require the procedure of transjugular intrahepatic portosystemic shunt (TIPS). Transjugular intrahepatic portosystemic shunt is an interventional radiology technique consisting of an artificial conduit from the portal to the systemic venous circulation created to resolve portal hypertension in order to manage these 2 complications, thus acting as a bridging therapy to liver transplantation.^1^

Hepatic encephalopathy (HE), a syndrome of altered neurological function due to metabolic deregulations, is yet another commonly seen complication in patients with cirrhosis. Though the pathogenesis of HE is very complex, but the key factor is increased concentration of gut-derived ammonia which bypasses the liver due to portosystemic shunts. The shunt connection between the splanchnic and systemic circulation formed in TIPS reduces the complications of variceal bleed and ascites but ironically shows increased association with the complication of HE. With 20%-31% of patients demonstrating either new or worsening HE after TIPS, it is considered a major drawback of this procedure as the ammonia-rich blood bypasses the liver detoxification process making its way straight to the systemic circulation and manifesting as HE.^2^

Sarcopenia, defined as loss of skeletal muscle mass, is a major cause of malnutrition seen in patients with cirrhosis. Almost 65%-90% of patients with cirrhosis have sarcopenia^3^ which adversely affects disease outcome in terms of increased risk of infections, development of hepatocellular carcinoma, poor quality of life, poor outcome of liver transplantation, and also increased hospital costs.^4,5^ In a failing liver, the muscle acts as an alternative site for ammonia detoxification.^6^ Reduction in muscle mass and even quality may lead to an increase in the circulating ammonia levels.^7^ Hyperammonemia per se reduces muscle protein synthesis and enhances muscle protein degradation.^8^ Hence, a reduced muscle mass may further augment the manifestation of post-TIPS HE in a cirrhotic patient.

Though a few studies have examined the relationship between sarcopenia and HE after TIPS, there are no Indian studies to suggest the same association. Hence, the present study was planned to investigate the prevalence of sarcopenia in patients who underwent TIPS and to study the effect of sarcopenia on the incidence of post-TIPS HE.

## MATERIALS AND METHODS

This study conforms to the provisions of the Declaration of Helsinki. Ethics Committee approval and informed consent were not taken due to the retrospective nature of the study as the data were collected from the hospital records.

### Study Population

**Figure 1.**
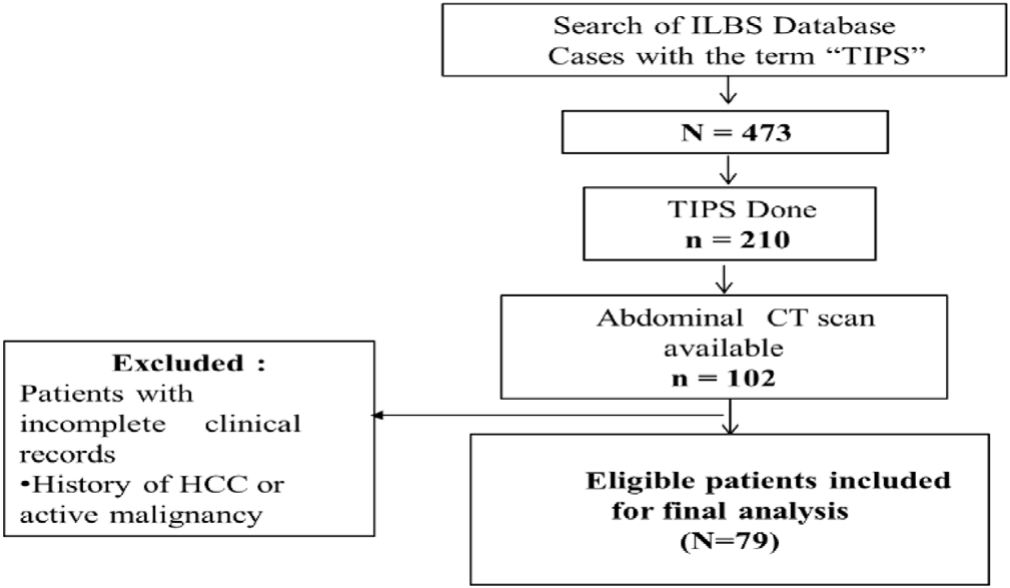
Schematic representation of data retrieval.

In this retrospective observational study, the clinical records of 210 cirrhotics who underwent TIPS at our institute from October 2010 to January 2018 were reviewed (Figure 1). Patients with age >18 years, irrespective of the etiology, who underwent TIPS at our center and had a computed tomography (CT) scan performed within 3 months prior to TIPS for diagnostic and/or interventional reasons were included in the study.

### Clinical and Laboratory Assessment

Data related to clinical characteristics, laboratory tests, and follow-up records were extracted from the hospital information system. Medical records were accessed using the hospital accession number from the database for demographic (age and gender), clinical (etiology, height, weight, an indication of TIPS, Child–Turcotte–Pugh (CTP) score, Model for End Stage Liver Disease score, and presence of HE), and radiological (CT scan) information at baseline and follow-up. Information on the day of TIPS was considered as a baseline and that on hospital re-admission was considered as a follow-up.

### Assessment of Hepatic Encephalopathy

The patient’s records were reviewed to look for episodes of HE after the TIPS procedure. Hepatic ncephalopathy was considered when there was a record of at least 1 hospital admission due to HE as defined by the West Haven criteria. Hepatic encephalopathy episodes after TIPS were categorized as HE absent (−), HE once (HE+), and more than once as multiple episodes of HE (HE++).

### Assessment of Sarcopenia by Computed Tomography Scan

**Figure 2.**
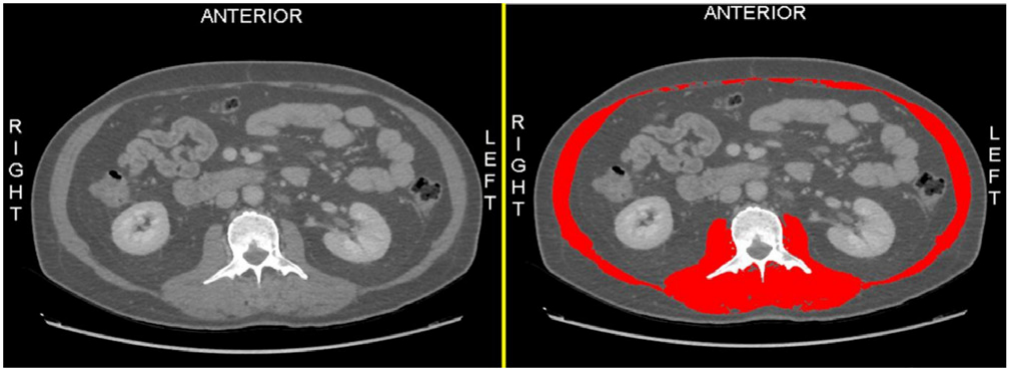
Computed tomography image for assessment of skeletal muscle area.

All CT scans were performed on Discovery 750 HD 64-row spectral CT scanner (GE, USA). Single-slice CT images at the third lumbar vertebra of the scans performed within 3 months prior to the TIPS procedure were analyzed using image analysis software Slice-O-MaticV4.3 (Tomovision Montreal, Canada). The skeletal muscle area (SMA, in cm^2^) was defined as the sum of the area of paraspinal, psoas, transverse abdominis, interior/exterior oblique, and rectus abdominis muscle at the level of the L3 region. Skeletal muscle was identified based on predefined CT density of −29 to +150 Hounsfield unit (HU).^9^ Muscle was measured using the paint tool of the software, with red color coding (Figure 2). The cross-sectional area was automatically computed by summing the tissue pixel and then multiplying it by the surface area of pixels. Skeletal muscle index (SMI) was then calculated as SMA (cm^2^)/height (m^2^). Sarcopenia was defined as SMI <50 cm/m^2^ in males and <39 cm/m^2^ in females.^10^

**Figure 3.**
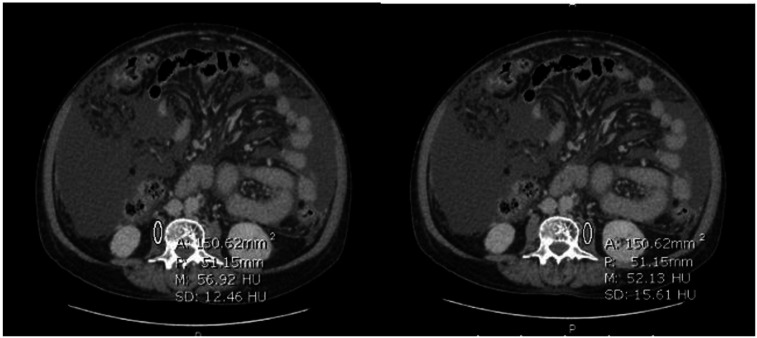
Computed tomography image for assessment of muscle density using region of interest.

Skeletal muscle density (SMD) was assessed by the mean radiological muscle attenuation of the psoas muscle at the L3 level. Bilateral psoas muscle attenuation expressed in the Hounsfield unit was measured using the hospital standard Picture archiving and communication system (PACS) imaging software. An oval region of interest of 1.5 cm^2^ was placed in the most homogenous area of the muscle (Figure 3).^11^ Measurements were obtained from each side, and the average psoas muscle attenuation value was calculated.

### Statistical Analysis

Data are presented as number (%), mean ± standard deviation, or median (range) as appropriate. Continuous variables were analyzed using Student’s *t*-test and categorical variables by Chi-square test. Wilcoxon rank-sum test was used for non-normal data. Besides this, univariate and multivariate logistic regression was also applied. *P* < .05 was considered statistically significant. All analyses were carried out using Statistical Package for the Social Sciences software version 22 (IBM Corp Ltd., Armonk, NY, USA).

## Results

### Patient and Disease Characteristics

Of the 210 patients who underwent TIPS, complete information was available for 79 patients. The demographic and clinical characteristics of patients included in the study are given in Table 1. Of these 79 patients, the majority were males (86%) with a mean age of 50.5 ± 11.2 years (28-70 years). The most common etiology of cirrhosis was alcohol in 44 (56%) followed by non-alcoholic steatohepatitis in 16 (20%), and others in 19 (24%). The most common indication for TIPS was refractory ascites in 56 (71%) followed by a variceal bleed in 23 (29%). The majority of patients who underwent TIPS were Child B cirrhotics (71%).

### Prevalence of Sarcopenia

Of the 79 patients who underwent TIPS, half (53%) of the patients had sarcopenia at baseline. The comparison of clinical, biochemical, and muscle parameters between sarcopenics and non-sarcopenics is shown in Table 2. Patients with sarcopenia had a lower body mass index (BMI) and more severe disease as reflected in their higher CTP score as compared to those without sarcopenia. Likewise, the serum ammonia level of sarcopenics was significantly higher as compared to the non-sarcopenics. Sarcopenics not only had a poor muscle mass (SMA) but also had a poor muscle quality as depicted by a significantly lower SMD.

### Post-transjugular Intrahepatic Portosystemic Shunt Hepatic Encephalopathy

Hepatic encephalopathy developed in 29 (37%) patients within a period of 1-9 months after the TIPS procedure. Table 3 shows the comparison of the clinical characteristics of patients with and without post-TIPS HE. The mean age, gender, BMI, and indication for TIPS were comparable between groups. Child–Pugh score and serum ammonia levels were significantly higher, while serum albumin was significantly lower in patients who developed post-TIPS HE as compared to those without HE. The incidence of post-TIPS HE was higher in patients with Child–Pugh B and C as compared to those with Child A.

Also, the prevalence of sarcopenia was significantly higher in patients who developed post-TIPS HE as compared to those who did not develop HE [69% vs. 44%, *P*  = .02; OR (95% CI): 2.8 (1.08-7.4), *P*  = .02], with sarcopenics having almost 3 times higher risk of developing HE.

Table 4 compares the frequency of post-TIPS HE in patients with and without sarcopenia. Multiple episodes of HE were significantly higher among sarcopenics [30.9% vs. 5.4%; OR (95%CI): 8.2 (1.68-40.5), *P*  = .009], thus taking the risk of multiple episodes of HE among sarcopenics to be 8 times higher than non-sarcopenics.

Further, on multivariate analysis (Table 5), age, serum albumin, and SMI were independently associated with post-TIPS HE in cirrhotic patients. Each unit increase in age increases the risk of post-TIPS HE by 5%, while each unit increase in serum albumin and SMI decreases the risk of post-TIPS HE by 84% and 7.5%, respectively.

## Discussion

Transjugular intrahepatic portosystemic shunt procedure, a therapeutic modality for treating cirrhotics with complications related to portal hypertension, is a double-edged sword with the qualms of development of HE after the procedure. A good number of patients who undergo TIPS procedure demonstrate new or worsening HE thereafter. Hepatic encephalopathy may be pure sequelae of liver failure or a result of a combination of both liver failure and portosystemic shunting.^1^ At our liver disease super specialty center, we conducted this retrospective study on patients who underwent TIPS for management of either refractory ascites or variceal bleed. Our assessment of sarcopenia in this group of patients revealed an alarmingly high prevalence of sarcopenia in almost half (53%) of these patients.

There exists a wide variation in the reported percentage of sarcopenia among cirrhotics.^7,12-16^ These wide variations could be due to the different diagnostic methods used for the assessment of sarcopenia like anthropometry,^15^ bioelectrical impedance analysis,^16^ and CT.^13^ In the present study, we used the gold standard technique of CT for muscle mass quantification to define sarcopenia. Notwithstanding the use of an advanced method of muscle quantification, our study falls short of the availability of complete information in all 210 patients who underwent TIPS; hence, the prevalence of sarcopenia could further be an underestimation.

Over the last decade, sarcopenia, the principal cause of malnutrition in cirrhosis, has been recognized as one of the additional factors responsible for the development of HE, thus further compounding the issue of post-TIPS HE. Patients with liver cirrhosis are prone to the development of skeletal muscle abnormalities because of reduced dietary intake, reduced hormone levels, inactivity, increased inflammation, and hyperammonemia. Hyperammonemia has a cumulative effect in accruing HE in a cirrhotic patient. The reduced capacity of the liver to detoxify ammonia elevates its concentrations which contributes to sarcopenia by interfering with the muscle protein synthesis pathways, that is, upregulating the myostatin secretion and stimulating muscle autophagy. Muscle, which potentially can play a compensatory role in ammonia detoxification, in the setting of sarcopenia, fails to detoxify ammonia, which in turn augments sarcopenia and further causes hyperammonemia; thus forming a vicious cycle.^17^ Likewise, the serum ammonia levels in the present study were commensurate with the incidence of post-TIPS HE. Not only was the prevalence of sarcopenia higher but also serum ammonia levels were significantly elevated in patients who developed HE after the TIPS procedure. Similar to our findings, a recent study done by Hanai et al^16^ also reported a higher prevalence of minimal HE (MHE) in patients with sarcopenia as compared to non-sarcopenics. Two more studies have also reported significantly higher ammonia levels in patients with muscle depletion or sarcopenia.^7,15^

Almost a 3 times higher risk of HE and 8 times higher risk of developing multiple episodes of post-TIPS HE in a cirrhotic patient, with pre-existing sarcopenia, poses an important query on pre-TIPS sarcopenia assessment and risk stratification prior to TIPS procedure. The artificial channel in TIPS created between the portal vein and the hepatic vein exposes the brain to the gut-derived ammonia and other toxic substances, thereby enhancing the probability of HE in a cirrhotic patient with an already failing liver.

Apart from the muscle mass, our study also highlights the association of muscle quality with the increased incidence of HE. A recent study by Bhanji et al^18^ studied the role of myosteatosis in the development of HE and found that myosteatosis and sarcopenia were both more frequent in patients who developed MHE. Merli et al^15^ also showed that the prevalence of both overt and MHE was significantly higher in patients with muscle depletion and those with decreased muscle strength as compared to those without these alterations. Muscle strength and myosteatosis are both considered markers of muscle quality, and hence, these studies prove that not only the reduced muscle mass but also muscle quality is the driving force for the development of HE in patients with liver cirrhosis.

Contrastingly, few observational studies have associated TIPS creation with reversal of muscle mass and improvement in body composition as well. Gioia et al^13^ reported significant improvement in SMI and muscle attenuation after a mean follow-up of almost 9 months following the TIPS procedure, with Psychometric Hepatic Encephalopathy Score and ammonia levels significantly improving in patients who showed >10% improvement in SMI. In a very recent study, TIPS creation was found to be associated with a significant increase in cross-sectional area and attenuation of truncal musculature after a median follow-up of 13.5 months.^19^ In yet another similar study, a significant increase in the total psoas and paraspinal muscle area and reduction in visceral fat volume have been observed in cirrhotic patients after the TIPS procedure.^20^

Though the literature does not elucidate very clearly about the molecular mechanisms behind the improvement in SMA after TIPS procedure, yet there are a few indications toward some plausible reasons like improved dietary intake after resolution of ascites^21^ and also improvement in the circulating levels of insulin-like growth factor 1 or even decrease in the muscle myostatin levels.^22^ Nevertheless, the improvement in muscle mass after TIPS procedure in these studies is quiet paradoxical to the previously reported literature with observations of increased incidence of post-TIPS HE associated with sarcopenia.

The present study did have its own limitations, like a relatively small sample size due to lack of complete information from the hospital records, missing information on patients having MHE or those with HE being admitted to some other hospital, and also the information related to precipitating factors such as electrolyte imbalance, constipation, etc., all these which are intrinsic to a retrospective study. Nonetheless, the present study clearly indicates that the risk of post-TIPS HE is manyfold higher in patients who have sarcopenia, and each unit increase in the skeletal muscle index decreases the risk of post-TIPS HE by 7.5%. With nearly half of the cirrhotics undergoing TIPS having sarcopenia, which reduces ammonia detoxification capacity and blatantly increases the risk of post-TIPS HE, a baseline sarcopenia risk assessment, appropriate stratification, and a prudent patient referral to the TIPS procedure are the need of the hour. Early and effective nutritional habilitation and physical training programs are very promising to get better outcomes of TIPS in terms of reduced incidence of HE and overall better quality of life.

## Figures and Tables

**Table 1.** Demographic and Clinical Characteristics of Patients with TIPS

**Parameter**	**Patients (n = 79)**
Males	68 (86)
Age (years)	50.5 ± 11.2
**Etiology**	
Alcohol	44 (56)
NASH	16 (20)
Others	19 (24)
**Indication for TIPS**	
Refractory ascites	56 (71)
Variceal bleed	23 (29)
Child–Pugh class (A/B/C)	3 (4)/56 (71)/20 (25)
Child–Pugh score	8.81 ± 1.23
MELD score	15.75 ± 6.38
Pre-TIPS HE	16 (20)

Data are expressed as number (%) and mean ± standard deviation.

TIPS, transjugular intrahepatic portosystemic shunt; NASH, non-alcoholic steatohepatitis; MELD, model for end stage liver disease; HE, hepatic encephalopathy.

**Table 2.** Comparison of Clinical and Biochemical Characteristics Between Patients With and Without Sarcopenia

**Parameter**	**Sarcopenia** **Present** **(n = 42)**	**Sarcopenia** **Absent** **(n = 37)**	*P*
Gender (male)	38 (90)	30 (81)	.33
Age (years)	51.8 ± 11.3	48.9 ± 11.1	.25
BMI (kg/m^2^)	25.1 ± 3.5	27.68 ± 5.1	**.01**
Etiology			
Alcohol	23 (55)	21 (57)	.18
NASH	6 (14)	10 (27)	
Others	13 (31)	6 (16)	
Indications for TIPS			
Refractory ascites	31 (74)	25 (68)	.62
Variceal bleed	11 (26)	12 (32)	
Child–Pugh class			
Child A	3 (7.1)	0 (0)	**.03**
Child B	25 (59.5)	31 (84)	
Child C	14 (33.4)	6 (16)	
Child–Pugh score	9.04 ± 1.32	8.5 ± 1.1	.06
MELD score	16.7 ± 7.5	14.6 ± 4.6	.17
Serum bilirubin (mg/dL)	1.7 (1.1-3.3)	1.5 (1.2-2.45)	.71
Serum albumin (g/dL)	2.6 ± 0.4	2.7 ± 0.5	.3
INR	1.5 ± 0.45	1.5 ± 0.3	.56
Serum creatinine (mg/dL)	1.2 ± 0.75	1.1 ± 0.9	.58
Serum sodium (mEq/dL)	133.5 ± 4.4	133.1 ± 6.3	.43
Serum ammonia (µg/dL)	157 ± 75.6	117.6 ± 46.4	**.03**
SMA (m^2^)	123.4 ± 17.3	150.3 ± 28.7	**<.001**
SMD (HU)	56.9 ± 11.2	64.4 ± 13.3	**.009**
HE (yes/no)	20/22	9/28	**.032**
Pre-TIPS HE (yes/no)	9/33	7/30	.503

Data are expressed as number (%), mean ± standard deviation, or median (min-max) as appropriate.

BMI, body mass index; NASH, non-alcoholic steatohepatitis; TIPS, transjugular intrahepatic portosystemic shunt; MELD, Model for End-Stage Liver Disease; INR, international normalized ratio; SMA, skeletal muscle area; SMD, skeletal muscle density; HU, Hounsfield Unit; HE, hepatic encephalopathy.

The bold value in table 2 represents the statistically significant values with a *P* < .05.

**Table 3.** Comparison of Clinical Characteristics Between Patients With and Without Post-TIPS HE

**Parameter**	**Post-TIPS HE Present** **(n = 29)**	**Post-TIPS HE Absent** **(n = 50)**	*P*
Gender (male)	23 (79)	45 (90)	.19
Age (years)	53 ± 10.8	48.9 ± 11.2	.09
BMI (kg/m^2^)	25.1 ± 4.37	27 ± 4.4	.08
Indications for TIPS			
Ascites	22 (76)	34 (68)	.6
Bleed	7 (24)	16 (32)	
Child–Pugh Score			
Child A	0 (0)	3 (6)	.17
Child B	19 (65.5)	37 (74)	
Child C	10 (34.5)	10 (20)	
Child–Pugh Score	9.34 ± 1.04	8.5 ± 1.23	**.003**
MELD score	17.5 ± 6.63	14.73 ± 6.08	.09
Serum bilirubin (mg/dL)	1.54 (0.85-2.83)	1.55 (1.2-2.8)	.5
Serum albumin (g/dL)	2.53 ± 0.5	2.81 ± 0.45	**.01**
INR	1.58 ± 0.48	1.5 ± 0.35	.41
Serum creatinine (mg/dL)	1.11 ± 0.69	1.14 ± 0.96	.86
Serum sodium (meq/dL)	133.6 ± 5.1	133.1 ± 5.5	.69
Serum ammonia (µg/dL)	177 ± 82.5	115 ± 40.5	**.009**
SMA (m^2^)	130 ± 28.7	139.5 ± 25.3	.13
SMI (cm^2^/m^2^)	45.5 ± 8.9	50.1 ± 8.6	**.03**
SMD (HU)	58.6 ± 12.9	61.4 ± 12.6	.34
Sarcopenia (yes/no)	20 (69)/9 (31)	22 (44)/28 (56)	**.02**

Data are expressed as number (%), mean ± standard deviation, or median (min-max) as appropriate.

TIPS, transjugular intrahepatic portosystemic shunt; HE, hepatic encephalopathy; BMI, body mass index; MELD, Model for End-Stage Liver Disease; INR, international normalized ratio; SMA, skeletal muscle area; SMI, skeletal muscle index; SMD, skeletal muscle density; HU, Hounsfield Unit; OR, odds ratio.

The bold value in table 3 represents the statistically significant values with a *P* < .05.

**Table 4.** Comparison of Frequency of Post-TIPS HE Between Sarcopenics and Non-sarcopenics

**Episode**	**Sarcopenia Present (42)**	**Sarcopenia Absent (37)**	**OR (95% CI)**	***P***
HE absent	22 (52.4)	28 (75.7)	1	-
HE once	7 (16.7)	7 (18.9)	1.27 (0.39-4.17)	.69
Multiple episodes	13 (30.9)	2 (5.4)	8.27 (1.69-40.57)	**.009**

Data are expressed as number (%). HE, hepatic encephalopathy; TIPS, transjugular intrahepatic portosystemic shunt; OR, odds ratio.

The bold value in table 4 represents the statistically significant values with a *P* < .05.

**Table 5.** Predictors of Post-TIPS HE in Patients with Cirrhosis

**Parameter**	**Univariate Regression Analysis OR (95% CI)**	***P***	**Multivariate Regression Analysis OR (95% CI)**	***P***
Gender (male)	0.43 (0.12-1.54)	.19		
Age (years)	1.04 (0.99-1.08)	.09	1.05 (1.001-1.11)	**.047**
BMI (kg/m^2^)	0.91 (0.81-1.013)	.08		
Indications for TIPS				
Ascites	1.48 (0.52-4.17)	.6		
Bleeding				
MELD score	1.07 (0.98-1.16)	.09		
Child–Pugh score	1.62 (1.15-2.28)	**.003**		
Serum bilirubin (mg/dL)	1.04 (0.92-1.16)	.5		
Serum albumin (g/dL)	0.27 (0.09-0.78)	**.01**	0.162 (0.05-0.56)	**.004**
INR	1.6 (0.51-4.92)	.41		
Serum sodium (meq/dL)	1.02 (0.93-1.11)	.69		
Serum ammonia (µg/dL)	1.02 (1.005-1.035)	**.009**		
SMA (cm^2^)	0.99 (0.97-1.004)	.13	0.925 (0.89-0.99)	**.017**
SMI (cm^2^/m^2^)	0.94 (0.89-0.99)	**.03**		
SMD (HU)	0.98 (0.94-1.02)	.34		

Data are expressed as OR (95%CI).

TIPS, transjugular intrahepatic portosystemic shunt; HE, hepatic encephalopathy; BMI, body mass index; MELD, Model for End-Stage Liver Disease; INR, international normalized ratio; SMI, skeletal muscle index; SMD, skeletal muscle density; HU, Hounsfield Unit; OR, odds ratio.

The bold value in table 5 represents the statistically significant values with a *P* < .05.
